# Unexpected Neuronal Protection of SU5416 against 1-Methyl-4-Phenylpyridinium Ion-Induced Toxicity via Inhibiting Neuronal Nitric Oxide Synthase

**DOI:** 10.1371/journal.pone.0046253

**Published:** 2012-09-25

**Authors:** Wei Cui, Zaijun Zhang, Wenming Li, Shinghung Mak, Shengquan Hu, Huan Zhang, Shuai Yuan, Jianhui Rong, Tony Chunglit Choi, Simon M. Y. Lee, Yifan Han

**Affiliations:** 1 Department of Applied Biology and Chemical Technology, Institute of Modern Medicine, The Hong Kong Polytechnic University, Hong Kong, China; 2 State Key Laboratory of Quality Research in Chinese Medicine, Institute of Chinese Medical Sciences, University of Macau, Macau, China; 3 Institute of New Drug Research, Guangdong Province Key Laboratory of Pharmacodynamic, Constituents of Traditional Chinese Medicine & New Drug Research, College of Pharmacy, Jinan University, Guang Zhou, Guangdong, China; 4 School of Chinese Medicine, the University of Hong Kong, Hong Kong, China; Universidad de Sevilla, Spain

## Abstract

SU5416 was originally designed as a potent and selective inhibitor of vascular endothelial growth factor receptor-2 (VEGFR-2) for cancer therapy. In this study, we have found for the first time that SU5416 unexpectedly prevented 1-methyl-4-phenylpyridinium ion (MPP^+^)-induced neuronal apoptosis in cerebellar granule neurons, and decreased 1-methyl-4-phenyl-1,2,3,6-tetrahydropyridine (MPTP)-induced loss of dopaminergic neurons and impairment of swimming behavior in zebrafish in a concentration-dependent manner. However, VEGFR-2 kinase inhibitor II, another specific VEGFR-2 inhibitor, failed to reverse neurotoxicity at the concentration exhibiting anti-angiogenic activity, strongly suggesting that the neuroprotective effect of SU5416 is independent from its anti-angiogenic action. SU5416 potently reversed MPP^+^-increased intracellular nitric oxide level with an efficacy similar to 7-nitroindazole, a specific neuronal nitric oxide synthase (nNOS) inhibitor. Western blotting analysis showed that SU5416 reduced the elevation of nNOS protein expression induced by MPP^+^. Furthermore, SU5416 directly inhibited the enzyme activity of rat cerebellum nNOS with an IC_50_ value of 22.7 µM. In addition, knock-down of nNOS expression using short hairpin RNA (shRNA) abolished the neuroprotective effects of SU5416 against MPP^+^-induced neuronal loss. Our results strongly demonstrate that SU5416 might exert its unexpected neuroprotective effects by concurrently reducing nNOS protein expression and directly inhibiting nNOS enzyme activity. In view of the capability of SU5416 to cross the blood-brain barrier and the safety for human use, our findings further indicate that SU5416 might be a novel drug candidate for neurodegenerative disorders, particularly those associated with NO-mediated neurotoxicity.

## Introduction

SU5416 (Semaxanib) was originally designed as a potent and selective inhibitor of vascular endothelial growth factor receptor-2 (VEGFR-2) for cancer therapy [1]. It occupies the ATP binding site of VEGFR-2, and thereby abolishes vascular endothelial growth factor (VEGF) signaling [1]. In the pre-clinical studies, SU5416 inhibits VEGF-dependent angiogenesis both *in vitro* and *in vivo* [2]. As the first VEGFR-2 inhibitor evaluated in clinical trial, SU5416 is well tolerated even at the concentration of 145 mg/m^2^ in patients with advanced malignancies in phase I clinical study [3]. It was found that SU5416 and 5-fluorouracil-leucovorin in combination showed better efficacy than standard 5-fluorouracil-leucovorin therapy in the pilot phase I/II study [4]. Nevertheless, test on this drug was discontinued for there were no significant clinical benefits in a randomized phase III trial [5]. Notably, SU5416 could be rapidly distributed to all organs, and accumulated in orthotopically implanted central nerve system (CNS) tumor model and in patients with refractory pediatric CNS tumors, suggesting that SU5416 could be delivered to the CNS by passing through the blood-brain barrier [6].

Parkinson’s disease (PD) is the second most common neurodegenerative disorder among the elderly worldwide [7], [8], [9]. Although the etiology of PD remains largely unknown, overproduction of nitric oxide (NO) is considered as a causative factor for the loss of dopaminergic neurons [10]. High levels of neuronal nitric oxide synthase (nNOS) are found in the nigrostriatal regions and basal ganglia of post-mortem PD brains and animals treated with 1-methyl-4-phenyl-1,2,3,6-tetrahydropyridine (MPTP), a PD-inducing neurotoxin [11]. On the other hand, transgenic mice that lack the nNOS gene are more resistant to MPTP than wild-type mice [12]. Selective nNOS inhibitors produce neuroprotective effects against MPTP both *in vitro* and *in vivo*. These results suggest that nNOS inhibitors might have therapeutic potential in the treatment of PD [10], [13], [14], [15].

Zebrafish serves as a good animal model to screen neuroprotective drugs [16], [17]. MPTP could induce the loss of dopaminergic neurons, decrease tyrosine hydroxylase expression in the posterior tuberculum of the ventral diencephalon, and subsequently impair motor behavior in zebrafish [17]. In this study, we first evaluated the neuroprotective activity of SU5416 against 1-methyl-4-phenylpyridinium ion (MPP^+^), the active metabolite of MPTP, induced neuronal death in primary cerebellar granule neurons (CGNs) and MPTP-induced dopaminergic neuronal loss and locomotion behavior impairment in zebrafish. We further demonstrated that SU5416 might exhibit neuroprotective effects by reducing nNOS expression and directly inhibiting nNOS activity rather than by suppressing angiogenesis as previously reported. The neuroprotective effect of SU5416 was also confirmed by experiments using short hairpin RNA (shRNA) knock-down of the NOS proteins in PC12 cells.

## Materials and Methods

### Ethics Statement of Animal Experiments

All rodent experiments were conducted according to the ethical guidelines of Animal Subjects Ethics Sub-committee (ASESC), the Hong Kong Polytechnic University; and the protocol was approved by ASESC, the Hong Kong Polytechnic University (permit number: 10/15). All surgeries were performed under sodium pentobarbital anesthesia, and all efforts were made to minimize animal suffering.

All zebrafish experiments were conducted according to the ethical guidelines of Institute of Chinese Medical Sciences (ICMS), University of Macau; and the protocol was approved by ICMS, University of Macau.

### Primary Cerebellar Granule Neuron Cultures

Rat CGNs were prepared from 8-day-old Sprague-Dawley rats (The Animal Care Facility, The Hong Kong Polytechnic University) as described in our previous publication [18]. Briefly, neurons were seeded at a density of 2.7×10^5^ cells/ml in basal modified Eagle’s medium (Invitrogen) containing 10% fetal bovine serum, 25 mM KCl, 2 mM glutamine, and penicillin (100 units/ml)/streptomycin (100 µg/ml). Cytosine arabinoside (10 µM) was added to the culture medium 24 hours after plating to limit the growth of non-neuronal cells. With the use of this protocol, more than 95% of the cultured cells were granule neurons.

**Figure 1:**
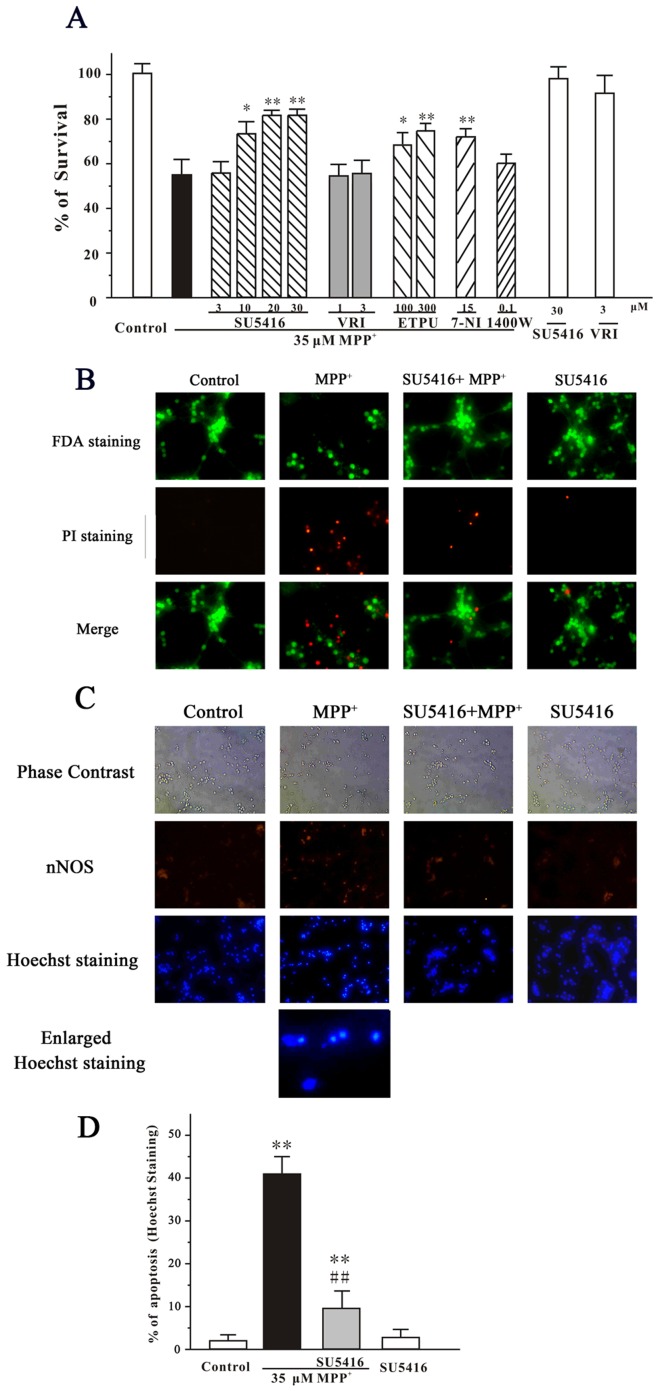
SU5416 prevents MPP^+^-induced apoptosis in a concentration-dependent manner. (A) SU5416, but not VRI, prevented MPP^+^-induced cell death in a concentration-dependent manner. CGNs were treated with SU5416, VRI, EPTU, 7-nitroindazole (7-NI), 1400 W or DMSO (vehicle control) at the indicated concentrations for 2 hours and then exposed to 35 µM MPP^+^. Cell viability was measured by MTT assay at 24 hours after MPP^+^ challenge. (B) SU5416 blocked neuronal loss induced by MPP^+^. CGNs were pre-incubated with or without 20 µM SU5416 and exposed to 35 µM MPP^+^2 hours later. At 24 hour after MPP^+^ challenge, CGNs were assayed with FDA/PI double staining. (C) SU5416 reversed the morphological alteration induced by MPP^+^. CGNs were pre-incubated with or without 20 µM SU5416 and exposed to 35 µM MPP^+^2 hours later. At 24 hour after MPP^+^ challenge, CGNs were assayed with nNOS and Hoechst double staining. (D) The number of apoptotic nuclei with condensed chromatin was counted from representative Hoechst staining photomicrographs and represented as a percentage of the total number of nuclei counted. Data, expressed as percentage of control, were the mean ± SEM of three separate experiments; *p<0.05 and **p<0.01 *versus* MPP^+^ group in (A) or *versus* control in (D); ^##^ *p*<0.01 *versus* MPP^+^ group in (D) (Turkey’s test).

### Measurement of Neurotoxicity

The percentage of surviving neurons in the presence of SU5416 and/or MPP^+^ was estimated by determining the activity of mitochondrial dehydrogenases with 3(4,5-dimethylthiazol-2-yl)-2.5-diphenyltetrazolium bromide (MTT) assay [13]. The assay was performed according to the specifications of the manufacturer (MTT kit I; Roche Applied Science). Briefly, the neurons were cultured in 96-well plates, 10 µl of 5 mg/ml MTT labeling reagent was added to each well containing cells in 100 µl of medium, and the plates were incubated at 37°C for 4 hours in a humidified incubator. After the incubation, 100 µl of the solvating solution (0.01 N HCl in 10% SDS solution) was added to each well for 16–20 hours. The absorbance of the samples was measured at a wavelength of 570 nm with 655 nm as a reference wavelength. Unless otherwise indicated, the extent of MTT conversion in cells exposed to MPP^+^ is expressed as a percentage of the control.

Cytotoxicity was determined by measuring the release of lactate dehydrogenase (LDH). Briefly, cells were precipitated by centrifugation at 500 g for 5 min at room temperature, 50 µl of the supernatants was transferred into new wells, and LDH was determined using the *in vitro* toxicology assay kit (Roche). The absorbance of the samples was measured at a wavelength of 490 nm with 655 nm as a reference wavelength.

**Figure 2:**
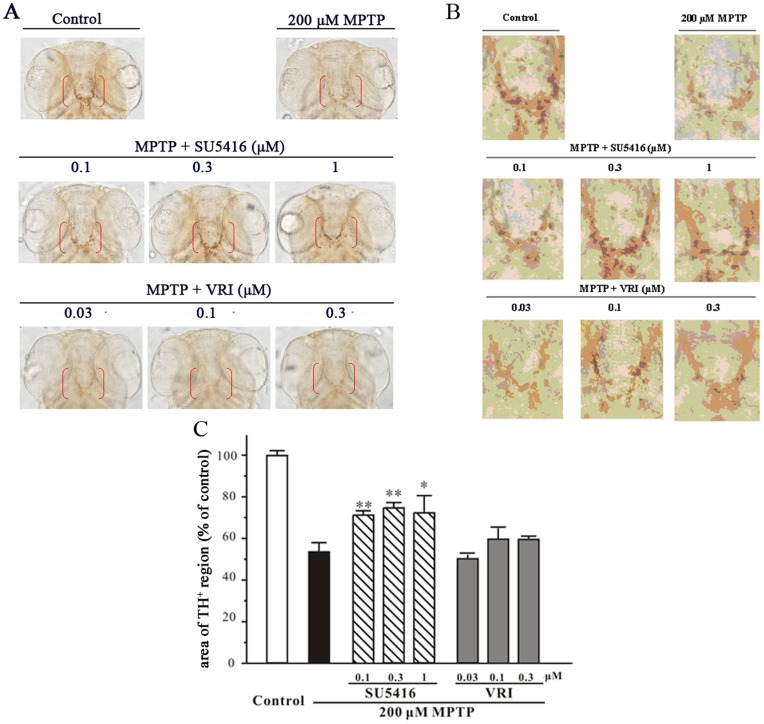
SU5416 protects against MPTP-induced TH+ region area decrease in zebrafish. One dpf zebrafish embryos were co-incubated with 200 µM MPTP and SU5416, VRI or 0.3% DMSO (vehicle control) at the indicated concentrations for 2 days. After treatment, zebrafish were collected to perform whole-mount immunohistochemistry. (A) Representative pictures of whole-mount immunostaining of zebrafish brain from different treatment groups. (B) Magnification of diencephalic area of zebrafish larval (indicated by red bracket in Fig. 2A). (C) Statistical analysis of TH^+^ region area in each treatment group (20 fish embryos per group). Data, expressed as percentage of control, were the mean ± SEM of three separate experiments; ^##^ *p*<0.01 *versus* control; *p<0.05 and **p<0.01 versus MPTP group (Turkey’s test).

### FDA/PI Double Staining Assay

Viable granule neurons were stained with fluorescein formed from fluorescein diacetate (FDA) by esterase in viable cells. Propidium iodide (PI) can penetrate cell membranes of dead cells to intercalate into double-stranded nucleic acids. Briefly, after incubation with 10 µg/ml of FDA and 5 µg/ml of PI for 15 min, the neurons were examined and images were acquired using UV light microscopy for comparison with photos taken under phase contrast microscopy.

### Hoechst Staining and Immunostaining

Chromatin condensation was detected by staining the cell nucleus with Hoechst 33342 as described in our previous publication [18]. CGNs (2.7×10^6^ cells) grown in a 35-mm dish were washed with ice-cold phosphate-buffered saline (PBS), fixed with 4% formaldehyde in PBS, membrane-permeabilized in 0.1% Triton X-100 and blocked in 1% BSA. Cells were then exposed to a primary nNOS antibody (Santa Cruz) overnight at 4°C followed by incubation at room temperature with an Alexa Fluor 488-conjugated secondary antibody. After immunostaining, cells were then stained with Hoechst 33342 (5 µg/ml) at 4°C for 5 min. Images were acquired using a fluorescence microscope at ×100 magnification.

To quantify the percentage of apoptotic nuclei in each group, photos of each dish (n = 3 dishes in each group for three independent experiments) were taken at five random fields and the numbers of apoptotic nuclei and total nuclei (n = 300) were counted, and the percentage of apoptotic nuclei was averaged.

**Figure 3:**
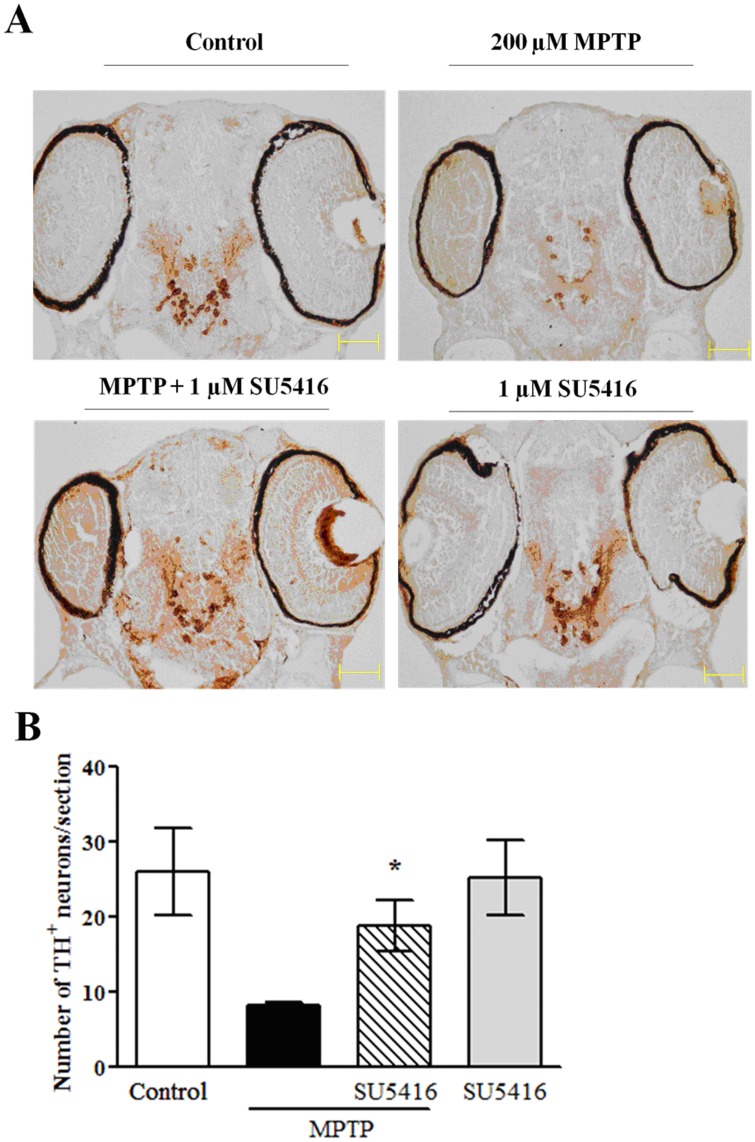
SU5416 increases the number of dopaminergic neurons in MPTP-treated zebrafish larval. One dpf zebrafish embryos were co-incubated with 200 µM MPTP and 1 µM SU5416 or 0.3% DMSO (vehicle control) for 2 days. After treatment, zebrafish were collected to perform paraffin-embedding, sectioning and immunostaining. (A) Representative picture of immunostaining of zebrafish section. (B) Statistical analysis of the number of TH-positive neurons in each treatment group (n = 12 fish/group). *p<0.05 *versus* MPTP group (Turkey’s test).

### Measurement of Intracellular NO

Intracellular NO was monitored with (4-amino-5-methylamino-2′,7′-difluorofluorescein) DAF-FM diacetate, a pH-insensitive fluorescent dye that emits increased fluorescence after reaction with an active intermediate of NO formed during the spontaneous oxidation of NO to NO_2_ [19]. DAF-FM solution was added to the culture medium (final concentration: 5 µM). After incubation for 30 min in a CO_2_ incubator, cultures were washed twice with PBS and incubated for another 30 min to allow complete de-esterification of the intracellular diacetate for strong fluorescence. The DAF-FM fluorescence in CGNs was quantified by a multi-detection microplate reader using excitation and emission wavelengths of 495 nm and 515 nm, respectively. The measured fluorescence values were expressed as a percentage of the fluorescence in the control cells.

### Western Blotting Analysis

Briefly, neurons were harvested in a cell lysis buffer. Protein was separated on SDS–polyacrylamide gel and transferred onto polyvinyldifluoride membranes. After membrane blocking, proteins were detected using primary antibodies. After incubation at 4°C overnight, signals were obtained after binding to chemiluminescent secondary antibodies. Blots were developed using an ECL plus kit (Amersham Bioscience, Aylesbury, UK) and exposed to Kodak autoradiographic films. All data were from three independent experiments and were expressed as the ratio to optical density (OD) values of the corresponding controls for statistical analyses.

**Figure 4:**
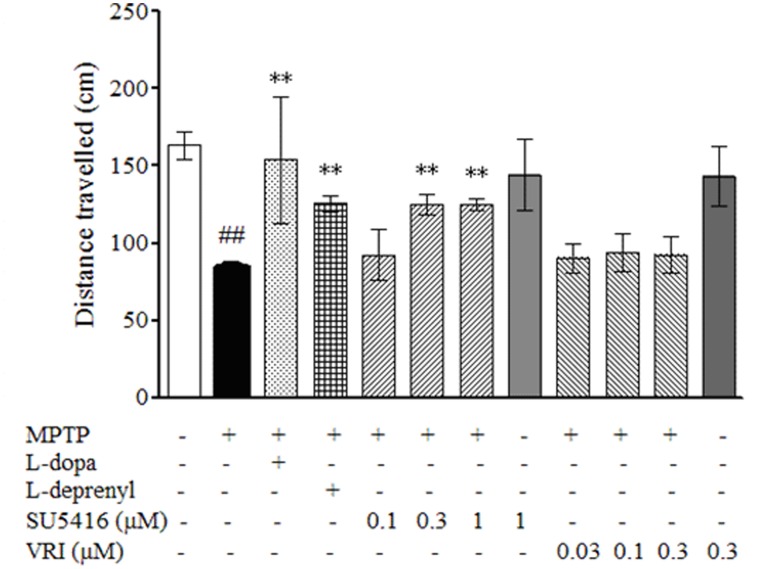
SU5416 attenuates the deficit of locomotion behavior on zebrafish larval induced by MPTP. One dpf zebrafish embryos were treated with 200 µM MPTP for 2 days, and then co-incubated with 10 µM MPTP and SU5416 or VRI at the indicated concentrations for 72 hours, and zebrafish larval co-treated with MPTP and 150 µM L-dopa or 20 µM L-deprenyl were used as positive controls. After treatment, zebrafish were collected to perform locomotion behavior test using Viewpoint Zebrabox system and total distances travelled in 10 min were calculated. Data, expressed as percentage of control, were the mean ± SEM of 12 fish larvae per group from 3-time independent experiments. ^##^ *p*<0.01 *versus* control group; **p<0.01 *versus* MPTP group (Turkey’s test).

### Maintenance of Zebrafish and Drug Treatment

Wild-type zebrafish (AB strain) and Tg(fli-1:EGFP) transgenic zebrafish were maintained as described in the Zebrafish Handbook [20]. Zebrafish embryos were generated by natural pair-wise mating (3–12 months old) and were raised at 28.5°C in embryo medium (13.7 mM NaCl, 540 µM KCl, pH 7.4, 25 µM Na_2_HPO_4_, 44 µM KH_2_PO, 300 µM CaCl_2_, 100 µM MgSO_4_, 420 µM NaHCO_3_, pH 7.4). Drugs were dissolved in DMSO and directly added into zebrafish embryo medium to treat fish without refreshing in 2–3 days (Final concentration of DMSO was always less than 0.5%, and showed no toxicity to zebrafish). Equal concentration of DMSO in embryo medium was used as vehicle control in each experiment.

### Exposure of Zebrafish to MPTP

Healthy zebrafish embryos were picked and dechorionated manually at 1 day post fertilization (dpf) then distributed into a 12-well plate with 20 fish embryos or a 6-well microplate with 30 fish embryos in each well. In pilot experiments, several doses of MPTP were added to embryo medium (final concentration from 50 to 800 µM) and 1 dpf fish embryo were treated for 48 hours, The optimal dose used (200 µM) induced a significant decrease in brain diencephalic dopaminergic neurons without any detectable systematic toxicity (data not shown). Thus subsequent studies were performed with 200 µM MPTP for whole-mount immunostaining and gene expression experiments.

**Figure 5:**
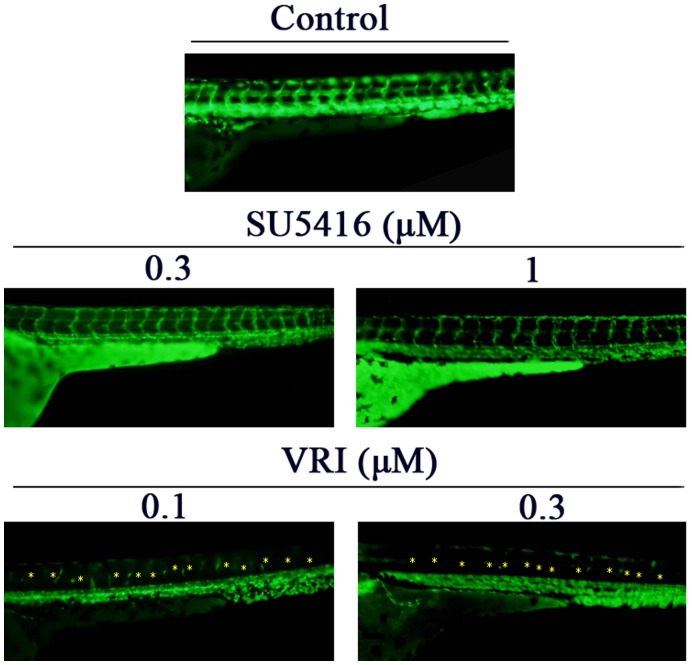
Anti-angiogenic effects of SU5416 and VRI in zebrafish. One dpf Tg*(fli-1:EGFP)* transgenic zebrafish embryos were treated with SU5416, VRI or DMSO (vehicle control) at the indicated concentrations for 2 days. After treatment, intersegmental-vessel formations were observed under fluorescence microscopy. Deficit of blood vessels was indicated by yellow asterisks.

**Figure 6:**
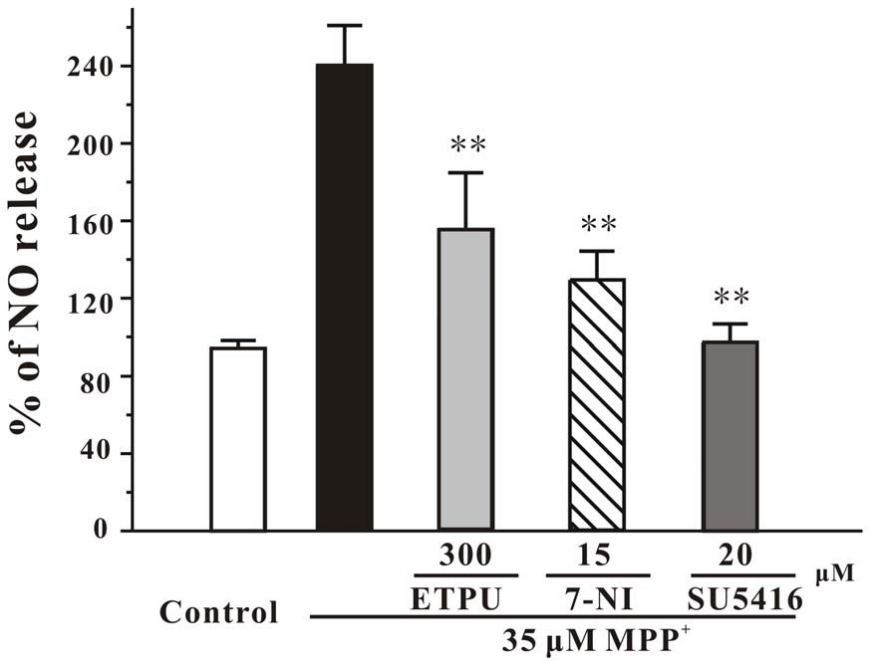
SU5416 reverses the elevated intracellular NO induced by MPP+ in CGNs. CGNs were pre-incubated with EPTU, 7-NI or SU5416 at the indicated concentrations for 2 hours, and exposed to 35 µM MPP^+^. Intracellular NO level was measured using DAF-FM diacetate as a probe at 8 hour after MPP^+^ challenge. Data, expressed as percentage of control, were the mean ± SEM of three separate experiments; **p<0.01 *versus* MPP^+^ group (ANOVA and Dunnett’s test).

Normally as late as 3 dpf, zebrafish larvae show very little spontaneous swimming, but by 5 dpf they spontaneously swim longer distances and independently search for food. The MPTP exposure therefore needs to last 5 days from 1 dpf. In pilot locomotion behavioral test, 3-day treatment starting from 1 dpf with 200 µM MPTP in embryo medium killed all the fish larvae, however, after 2-day treatment at 1 dpf with 200 µM MPTP then withdraw 3 days, and the deficit behavior recovered at 6 dpf. Finally, the optimal MPTP exposure was after 2-day treatment starting from 1 dpf with 200 µM MPTP, zebrafish larvae were maintained in embryo medium containing 10 µM MPTP for another 3 days, the swimming distance significantly decreased and without any detectable systematic toxicities. Thus subsequent locomotion behavioral studies were performed with 200 µM MPTP for 2-day treatment at 1 dpf then replacing with media containing 10 µM MPTP for another 3-day incubation.

### Whole-mount Zebrafish Immunostaining with Antibody against Tyrosine Hydroxylase

Whole-mount immunostaining in zebrafish was performed as before [21]. Briefly, zebrafish were fixed in 4% paraformaldehyde (wt/vol in PBS) for 5 h at room temperature or overnight at 4°C, washed with PBS 3 times, then kept in absolute ethanol at −20°C to dehydrate for at least 2 h or up to 1 week. Fixed samples were bleached in 10% H_2_O_2_ then blocked (2% lamb serum and 0.1% BSA in PBST) for 1 h at room temperature. A mouse anti-tyrosine hydroxylase (TH) monoclonal antibody (Millipore, USA) was used as the primary antibody and incubated with the sample overnight at 4°C. On the next day, samples were washed 6 times with PBST (30 min each wash), followed by incubation with secondary antibody according to the method provided by the Vectastain ABC kit (Vector Laboratories, USA). After staining, zebrafish were flat-mounted with 3.5% methylcellulose and photographed. Semi-quantification of area of TH^+^ region was assessed by an investigator blinded to the drug treatment history of zebrafish using Image-Pro Plus 6.0 software (Media Cybernetics, Silver Spring, MD, USA). Results were expressed as percentage of area of TH^+^ region in untreated control group.

**Figure 7:**
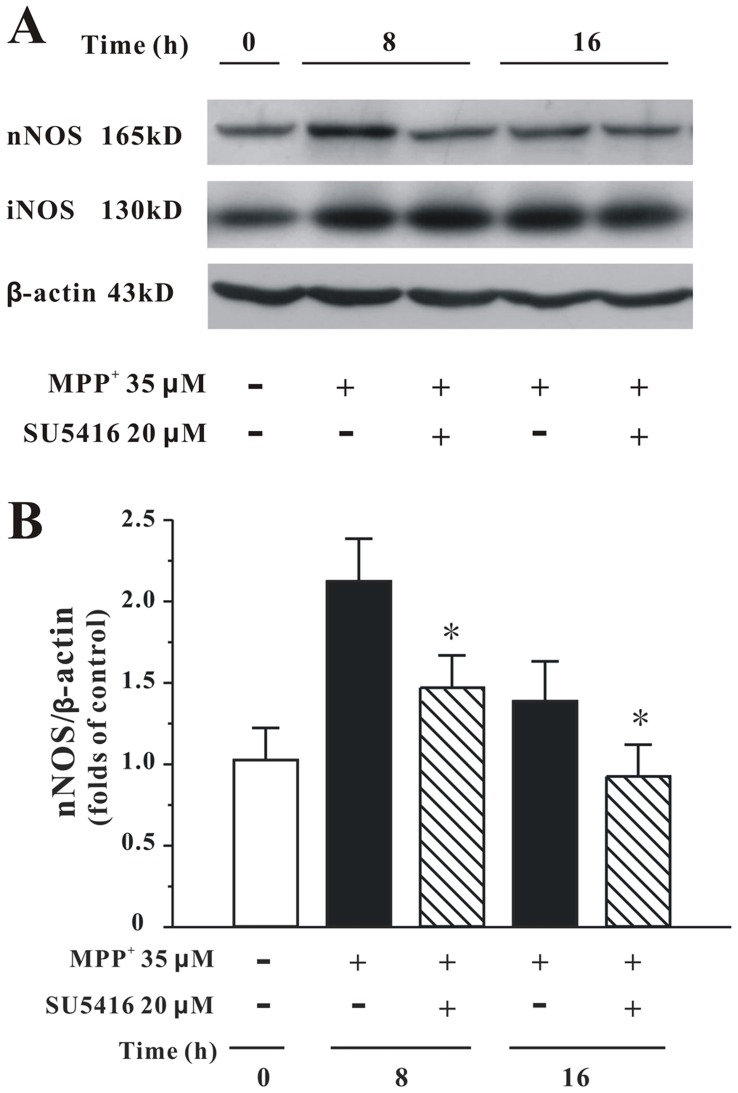
SU5416 reduces the expression of nNOS protein elevated by MPP+ in CGNs. (A) CGNs were pre-treated with 20 µM SU5416 or DMSO (vehicle control) for 2 hours, and then treated with 35 µM MPP^+^ for various durations as indicated. The total proteins were extracted for Western blot analysis with specific iNOS, nNOS and β-actin antibodies. (B) Statistical analysis of nNOS expression in each treatment group. Data are expressed as the ratio to OD values of the corresponding controls. Data, expressed as percentage of control, were the mean ± SEM of five separate experiments; *p<0.05 *versus* MPP^+^ group at the same time (Turkey’s test).

### Paraffin-embedding, Serial Sectioning and Immunostaining of Zebrafish Larval

After drug treatment, fixation of zebrafish larval was performed as the procedure in whole-mount immunostaining. We then mounted fixed specimens on 1% agarose blocks in a common linear plane to ensure that the microtome blade passes through each specimen simultaneously. The specimen-containing agarose was converted into a sectionable paraffin wax block and conducted processes as described by Sabaliauskas *et al.* [22]. Consecutive coronal sections were cut 5 µM thick using a rotary microtome (Leica RM2235, Germany) and mounted on microscope slides. Immunostaining of zebrafish larval sections was performed as previously described [23] with minor modifications. Paraffin sections were deparaffinized in xylene, hydrated in graduated alcohol solutions and incubated for 30 min in 3% H_2_O_2_ in PBS to inactivate endogenous peroxidases. Following antigen retrieval in citrate buffer for 15 min in a microwave oven, sections were blocked at room temperature with 10% horse serum for 1 hour. Sections were reacted overnight at 4°C with rabbit anti-mouse TH polyclonal antibody (Millipore, USA) at 1∶400 dilutions in immunostaining primary antibody dilution buffer (Beyotime, China). For detection of primary antibody, the EnVision Detection kit (Gene Tech., Shanghai, China) was used. Detection was done by the appropriate second antibody with peroxidase conjugate and DAB substrate. Finally, sections were coverslipped with neutral balsam. The results were analyzed by counting the numbers of TH-positive cells at ×20 magnifications on a stereomicroscope (BX51, Olympus Corp. Japan). TH-positive cells in 3 matched sections of each zebrafish were counted and averaged. 12 fish per treatment group were employed. The average number of TH-positive cells per section was used to represent dopaminergic neuron livability.

**Figure 8:**
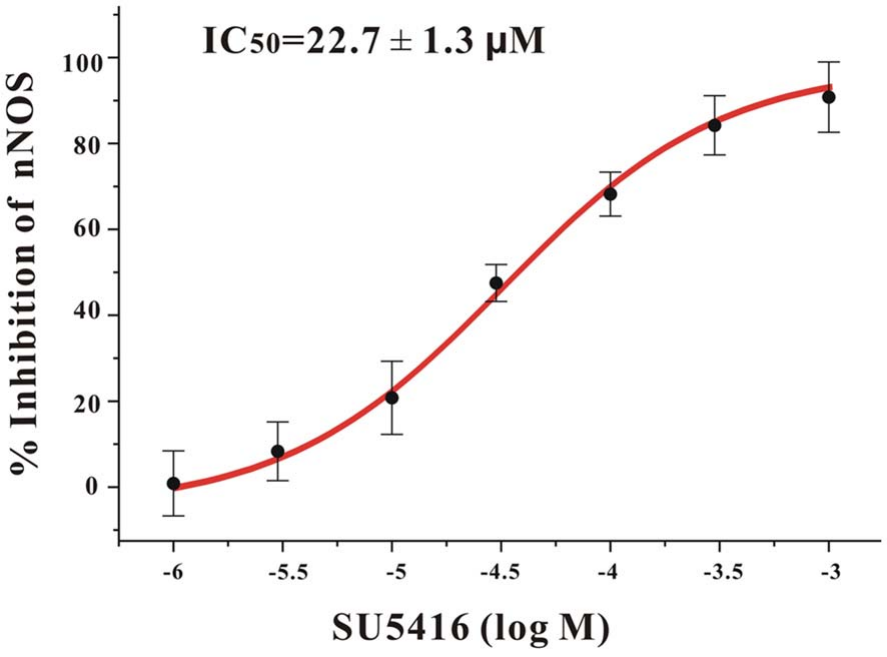
SU5416 directly inhibits nNOS enzyme activity in a concentration-dependent manner. The inhibitory effects of SU5416 on rat cerebellum nNOS were shown in the graph. The IC_50_ value was also indicated. Each individual point was an average from three independent experiments.

### Locomotion Behavioral Test of Zebrafish

After drug treatment, zebrafish larvae at 6 dpf were transferred into 96-well plates (1 fish/well and 12 larvae/group). The larvae were discarded due to excessive stress reaction to the handling and monitoring of behavior (such as rapid and disorganized swimming or immobility for 2 min). The experiments were performed in a calm sealed area. The larvae were allowed to habituate to the new environment for 30 min. Swimming behavior was monitored by an automated video tracking system (Viewpoint, ZebraLab, LifeSciences). The 96-well plates and camera were housed inside a Zebrabox and the swimming pattern of each fish was recorded for 10 min and for 3 times, once every other 10 min. The total distance moved was defined as the distance (in cm) that the fish had moved during one session (10 min).

### Morphological Observation of Zebrafish

After drug treatment, zebrafish were removed from the microplate and observed for gross morphological changes of blood vessel under a fluorescence microscope (Olympus IX81 Motorized Inverted Microscope, Japan) equipped with a digital camera (DP controller, Soft Imaging System, Olympus). Images were analyzed with Axiovision 4.2 and Adobe Photoshop 7.0.

### In vitro nNOS Activity Assay

Rat cerebellum nNOS was from Calbiochem. NOS activity was determined by monitoring the conversion of L-[^3^H]arginine to [^3^H]citrulline following the instructions provided by the kit (Calbiochem). The reaction mixture contained a final volume of 40 µl with 25 mM Tris-Cl at pH 7.4, 3 µM tetrahydrobiopterin, 1 µM FAD, 1 µM FMN, 1 mM NADPH, 0.6 mM CaCl_2_, 0.1 µM calmodulin, 2.5 µg of pure NOS enzyme, 5 µl L-[^3^H]arginine (Perkin Elmer, Waltham, MA, USA), and different concentrations of the tested reagents. The reaction mixture was incubated at 22°C for 45 min. The reaction was quenched by adding 400 µl of stopping buffer (50 mM HEPES, pH 5.5, and 5 mM EDTA). Unreacted L-[^3^H]arginine was then trapped by 100 µl of equilibrated resin in a spin cup followed by centrifugation at 13,200 rpm for 30 s.

### shRNA Design

ShRNA against rat nNOS was designed according to a previous publication [24]. Briefly, the SiRNA sequence GCACUGGUGGAGAUCAACA, which corresponds to exon 10 of the rat nNOS (GenBank Accession No. NM_052799), was used to generate shRNA. Oligonucleotides that contained the sense and antisense sequences of the siRNA target of interest flanking a standard hairpin loop sequence (TTCAAGAGA) were synthesized. Sense and antisense oligonucleotides were then annealed and cloned into pG418-GFP vector to express shRNA directed against nNOS under the control of the U6 promoter (GenePharma, Shanghai, China). A negative control shRNA (ShNC) with the same nucleotide composition but lacks significant sequence homology to the genome was also used in the experiments.

### Cell Transfection

PC12 pheochromocytoma cells were cultured in medium that consisted of DMEM, 10% heat-inactivated horse serum, 5% fetal bovine serum, 100 U/ml penicillin, and 100 µg/ml streptomycin in a 37°C, 5% CO_2_ incubator. 2.0×10^5^ cells were transfected with 3 µg indicated plasmids by using Lipofectamine 2000 (Invitrogen) according to the manufacturer’s instructions. Selection media that contained 100 µg/ml G418 (Sigma) were added to the cells 24 hours after transfection.

**Figure 9:**
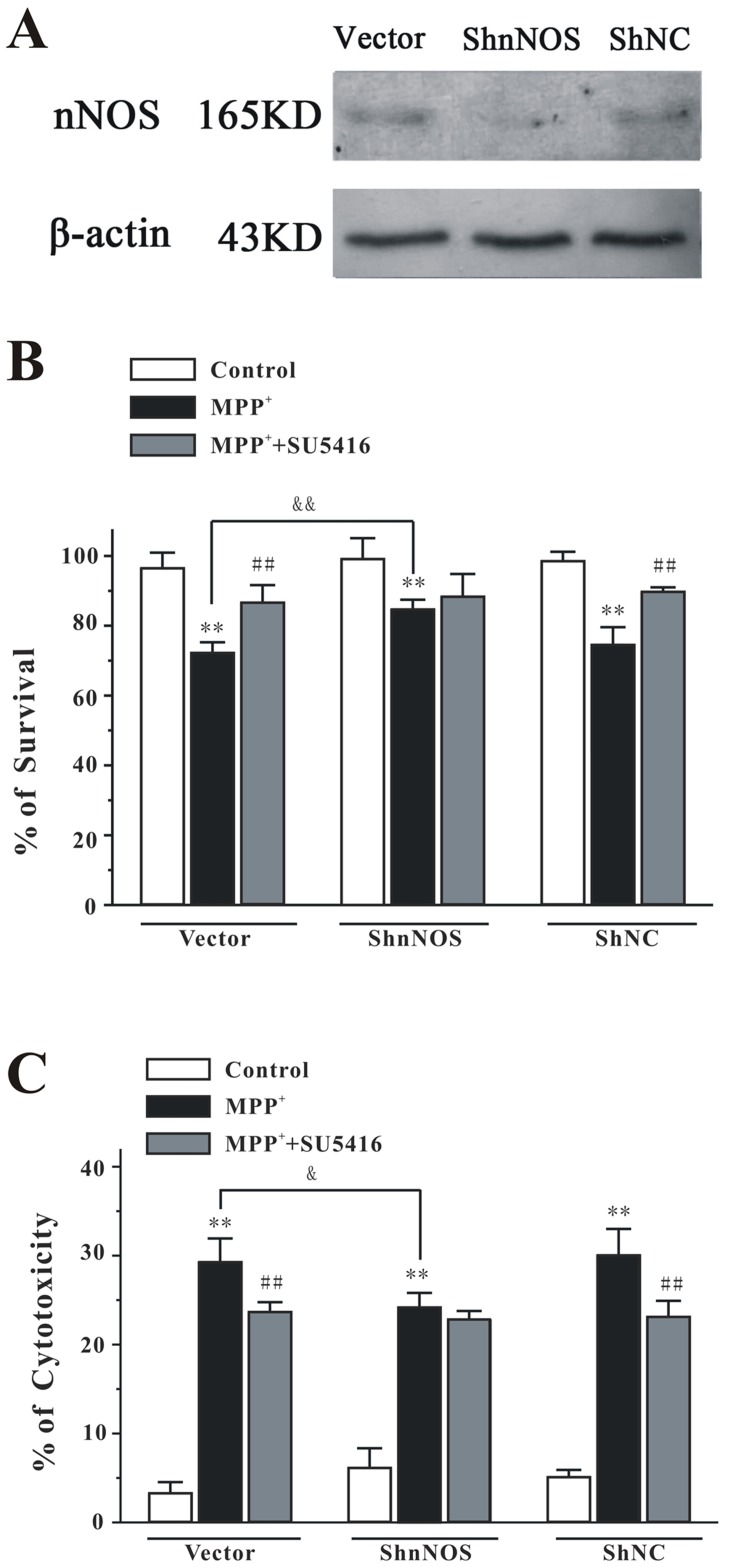
nNOS depletion abolishes the neuroprotective effects of SU5416 against MPP+-induced neuronal death in PC12 cells. (A) PC12 cells were transfected with pG418-GFP plasmid (vector), pG418-GFP plasmid encoding nNOS ShRNA (ShnNOS) and pG418-GFP plasmid encoding negative control ShRNA (ShNC). The levels of nNOS and β-actin in the cell lysates were analyzed by Western blotting assay by using specific antibodies. (B, C) nNOS depletion abolished the neuroprotective effects of SU5416 against MPP^+^-induced neuronal death in PC12 cells. PC12 cells transfected with vector, ShnNOS, or ShNC were treated with 20 µM SU5416 for 2 hours and then exposed to 1 mM MPP^+^. Cell viability (B) and cytotoxicity (C) were measured at 24 hours after MPP^+^ challenge by MTT and LDH assays, respectively. Data were the mean ± SEM of three separate experiments; **p<0.01 *versus* control; ^##^ *p*<0.01 *versus* MPP^+^ group; ^&^ *p*<0.05 and ^&&^ *p*<0.01 *versus* MPP^+^ vector group (Turkey’s test).

### Data Analysis and Statistics

Data are expressed as the means ± SEM, and statistical significance was determined by analysis of variance with Dunnett’s test in the case of multiple comparisons with control or Turkey’s test. Differences were accepted as significant at *p*<0.05.

## Results

### SU5416 Prevented MPP^+^-induced Neuronal Apoptosis in a Concentration-dependent Manner

After cultured for 8 days *in vitro*, CGNs were pre-treated with SU5416 at the concentrations of 3, 10, 20 or 30 µM for 2 hours, and then treated with 35 µM MPP^+^ for another 24 hours. Cell viability was measured using the MTT assay. It was found that SU5416 prevented 35 µM MPP^+^-induced cell death in a concentration-dependent manner (Fig. 1*A*). However, treatments with 30 µM SU5416 alone for 26 hours did not produce any cell proliferative or cytotoxic effects. VEGFR-2 kinase inhibitor II (VRI), another specific VEGFR-2 inhibitor with an IC_50_ value of 70 nM, was also tested in this model. Interestingly, VRI at 1 and 3 µM failed to block neuronal loss *in vitro* (Fig. 1*A*).

To further characterize the effects of SU5416 on the neurotoxicity of MPP^+^, CGNs were pretreated with 20 µM SU5416 and exposed to 35 µM MPP^+^ for 2 hours. The neurons were examined by FDA/PI double staining. It was found that SU5416 significantly blocked the loss of neurons and reversed the morphological alteration, including unhealthy bodies and broken extensive neuritic network, induced by MPP^+^ (Figs. 1*B and 1C*). According to the counts of apoptotic bodies stained by Hoechst 33342, SU5416 significantly reversed neuronal apoptosis induced by MPP^+^ (Figs. 1*C and 1D*).

NO is implicated in the neurotoxicity of MPP^+^ [12], [25]. To investigate whether NO was involved in our neuronal apoptosis model, nNOS immunostaining and NOS inhibitors were used to treat neurons for 2 hours prior to the addition of MPP^+^. It was observed that there were nNOS-positive neurons in our CGNs (Fig. 1*C*). Moreover, a pan-NOS inhibitor 2-ethyl-2-thiopseudourea (EPTU, IC_50_ values of 0.017 µM for iNOS. and 0.036 µM for nNOS) prevented MPP^+^-induced neuronal death in CGNs (Fig. 1*A*). The roles of NOS iso-enzymes were also examined by using specific inhibitors. We found that the specific nNOS inhibitor 7-nitroindazole (IC_50_ values of 0.7 µM for nNOS, and 20 µM for iNOS) protected against neuronal apoptosis in our model, whereas iNOS inhibitor 1400 W (IC_50_ values of 0.007 µM for nNOS, and 2 µM for iNOS) did not show protection (Fig. 1*A*).

### SU5416 Prevented MPTP-induced Neurotoxicity in Zebrafish

To assess the neuroprotective potential of SU5416 *in vivo*, zebrafish embryos at 1 dpf were exposed to 200 µM MPTP for 2 days, and the dopaminergic system in the brain of zebrafish was then examined by whole-mount immunostaining with specific antibody against TH. After MPTP treatment, the area of TH-immunoreactive regions observed in the diencephalons of zebrafish (indicated by red brackets) were decreased dramatically (Figs. 2*A and 2B*). Importantly, SU5416 (0.1–1 µM) significantly prevented the decrease in the area of TH^+^ region induced by MPTP. In contrast, VRI (0.03–0.3 µM) could not prevent MPTP-induced decrease in TH^+^ region area in zebrafish (Fig. 2). Both drugs at higher concentration, SU5416 at 10 µM and VRI at 3 µM, showed toxicity to zebrafish (data not shown).

To further confirm the protective effect of SU5416 against MPTP-induced dopaminergic neurotoxicity and to accurately observe changes of dopaminergic neurons in zebrafish, paraffin-embedding, serial sectioning and immunostaining of zebrafish larval were performed. TH-positive neuron count showed MPTP treatment significantly decreased the number of dopaminergic neurons, and 1 µM SU5416 co-treatment obviously prevented the loss of dopaminergic neurons (Fig. 3). SU5416 treatment alone did not notably alter the number of dopaminergic neurons.

As shown in Fig. 4, the total distance travelled by the zebrafish larvae decreased significantly after exposure to MPTP. SU5416 but not VRI ameliorated the MPTP-induced deficit of swimming behavior, which was also rescued by treatment with L-dopa and L-deprenyl (selegiline) as positive controls. Neither SU5416 nor VRI treatment alone notably altered the swimming behavior of normal zebrafish larvae (Fig. 4).

### The Neuroprotective Effects of SU5416 were not Directly Correlated with its Anti-angiogenic Action

We further determined if SU5416 at particular concentration ranges exhibited any anti-angiogenic activities in Tg*(fli1:EGFP)* transgenic zebrafish embryos. Owing to the genetic addition of a GFP gene under the control of the *fli-1* promoter, the fli-1 promoter activity in the endothelial cells of such zebrafish model can be directly observed using fluorescence microscopy. As shown in Fig. 5, VRI (0.1–0.3 µM) inhibited the formation of intersegmental-vessels in zebrafish larvae, whereas SU5416 (0.3–1 µM) did not show this activity.

### SU5416 Prevented MPP^+^-induced Increase of Intracellular NO Release

To investigate whether SU5416 protected against MPP^+^-induced neurotoxicity from acting on NO release, an intracellular NO measurement was used in our study. When CGNs were treated with SU5416 and MPP^+^ simultaneously, SU5416 antagonized the stimulatory effect of MPP^+^ on the NO production with an efficacy similar to 7-nitroindazole (15 µM) (Fig. 6). Our results suggest that the neuroprotection of SU5416 against MPP^+^-induced neuronal loss might be mediated by decreasing NO neurotoxicity, probably by inhibiting nNOS over-activation.

### SU5416 Reduced MPP^+^-increased Expression of nNOS Protein

To determine the effect of SU5416 on the protein expressions of nNOS and iNOS in CGNs, Western blotting analysis was used. As shown in Fig. 7, SU5416 at 20 µM reversed the increased expression of nNOS by MPP^+^. However, SU5416 at the same concentration could not affect the elevated expression of iNOS by MPP^+^ (Fig. 7A).

### SU5416 Directly Inhibited the Activity of nNOS

Furthermore, to investigate whether SU5416 also affected the activity of nNOS, an *in vitro* NOS activity assay was used in this study. It was found that SU5416 directly inhibited rat cerebellum nNOS in a concentration-dependent manner with an IC_50_ value of 22.7 µM (Fig. 8). These results suggest that SU5416 not only decreased the expression of nNOS, but also directly inhibited the activity of nNOS.

### nNOS Depletion Abolished the Neuroprotective Effects of SU5416

To explore if the neuroprotective effects of SU5416 mainly act through nNOS, we investigated the neuroprotection of SU5416 against MPP^+^-induced neurotoxicity in ShRNA-mediated nNOS knockdown PC12 cells. Western blot analysis showed that nNOS ShRNA (ShnNOS) caused a reduction in nNOS protein level, whereas the negative control ShRNA (ShNC) had no effect on nNOS protein level (Fig. 9*A*). Analyses of cell viability and cytotoxicity revealed that nNOS depletion resulted in a significant decrease in MPP^+^-induced cell death (Figs. 9*B and 9C*). We found that, in contrast to the neuroprotection effects of SU5416 observed in the vector or in the ShNC treated PC12 cells, SU5416 in nNOS knockdown PC12 cells was no longer able to inhibit MPP^+^-induced cell death (Figs. 9*B and 9C*). These results provided direct supporting evidence that the neuroprotective effects of SU5416 mainly act through the nNOS enzyme.

## Discussion

SU5416 is the first clinically evaluated VEGFR-2 inhibitor. Although previous clinical trails did not recommend SU5416 as an anti-cancer drug, SU5416 appeared to be safe in human use. In this study, we demonstrated for the first time that SU5416 was a promising neuroprotectant against MPP^+^/MPTP-induced neurotoxicity both *in vitro* and in zebrafish. Our results further revealed that the neuroprotection of SU5416 was not closely correlated with its anti-angiogenic action, but via attenuating NO-mediated neurotoxicity, possibly by both decreasing nNOS protein expression and directly inhibiting nNOS enzyme activity.

SU5416 was originally designed as a potent VEGFR-2 inhibitor with an IC_50_ value of 0.39 µM against the cellular VEGFR-2 tyrosine kinase activity [1]. To clarify whether its neuroprotection was due to the inhibition of VEGFR-2-dependent angiogenesis, another potent and selective VEGFR-2 inhibitor VRI was assessed in parallel. Interestingly, SU5416 at its neuroprotective concentration did not inhibit angiogenesis, whereas VRI did not prevent neuronal loss at the concentration in which it showed potent anti-angiogenic activity. These results suggest that the neuroprotection of SU5416 was not closely correlated with its anti-angiogenic property. Previous studies also showed that the activation of VEGFR-2 promoted neuronal survival by regulating phosphoinositide 3-kinase (PI3-K)/Akt and extracellular signal-regulated kinase (ERK) pathways [26]. The PI3-K/Akt signaling pathway is a pro-survival pathway, whereas the ERK pathway is a pro-apoptotic pathway in MPP^+^-induced neuronal apoptosis in CGNs [27]. To examine whether SU5416 acts on down-stream pathways of VEGFR-2, such as the ERK and Akt pathways, to protect against MPP^+^-induced neurotoxicity, we tested the activities of phospho-Akt (pAkt) and phospho-ERK (pERK) in Western blot assay (data not shown). Our results show that SU5416 could neither inhibit the activation of pro-apoptotic ERK pathway, nor reverse the decrease of pro-survival Akt pathway, suggesting that the neuroprotective effect of SU5416 is independent from the regulation of the PI3-K/Akt and ERK pathways.

It is well-known that NO is a central pro-apoptotic factor mediating the neurotoxicity of MPP^+^/MPTP both *in vitro* and *in vivo* [25], [28]. Intracellular NO could form peroxynitrite by reacting with superoxide, a kind of reactive oxygen species overproduced in MPP^+^-treated neurons. The resulted peroxynitrite could directly cause neuronal loss by nitrating cellular protein, damaging DNA and disrupting mitochondria [29]. We found that SU5416 decreased the elevated level of intracellular NO induced by MPP^+^, which suggested that SU5416 might exert its neuroprotective effects by regulating NO formation. Endogenous NO is mainly produced by a family of NOS enzymes. Three isoforms of NOS, namely nNOS (NOS-1), iNOS (NOS-2) and endothelial NOS (eNOS, NOS-3), have been identified so far. It is noteworthy that ablation of eNOS has no bearing on MPP^+^-induced neurotoxicity [28]. In the present study, we demonstrated that MPP^+^ increased the expression of nNOS, but not iNOS in CGNs. nNOS inhibitor 7-nitroindazole, but not iNOS inhibitor 1400 W, reduced MPP^+^-induced neuronal loss. These results suggested that MPP^+^-induced neurotoxicity was mainly mediated by the over-activation of nNOS, and SU5416 prevented neurotoxicity possibly by targeting nNOS.

According to Western blotting analysis, SU5416 reduced MPP^+^-elevated protein expression of nNOS. By assaying *in vitro* NOS activity, we further demonstrated that SU5416 directly inhibited the activity of nNOS with IC_50_ value of 22.7 µM. Most importantly, nNOS depletion abolished the neuroprotective effects of SU5416 against MPP^+^-induced neuronal death. These results strongly suggested that SU5416 most likely prevented NO-mediated neurotoxicity via both inhibiting the activity and decreasing the expression of nNOS. Although the precise mechanisms underlying the decrease of nNOS expression induced by SU5416 is still unclear, a recent study demonstrated that SU5416 could down-regulate the PI3K/Akt signaling pathway, a critical mediator in the activation of nNOS gene transcription induced by retinoic acid [30], suggesting that SU5416 might reduce the protein expression of nNOS via down-regulating the Akt pathway.

The production of neurotoxic NO by nNOS is implicated in many neurodegenerative disorders. Selective nNOS inhibitors may thus have therapeutic potential in treating neurodegenerative disorders by preventing neuronal death [31]. In this study, we have shown for the first time that SU5416 possesses neuroprotective potential against MPP^+^/MPTP-induced neurotoxicity both *in vitro* and *in vivo*. We have also demonstrated that SU5416 prevents neurotoxicity by reducing nNOS protein expression and directly inhibiting the enzyme activity of nNOS. In view of the capability of SU5416 to cross the blood-brain barrier and the safety for human use, our findings further indicate that SU5416 might be a novel drug candidate for neurodegenerative disorders and CNS cancers, particularly those associated with NO-mediated neurotoxicity.
